# Arthroscopic Bankart Repair Using 1 Anterior Portal Has a Shorter Surgical Time and Comparable Clinical Results With the Standard 2-Portal Technique

**DOI:** 10.1016/j.asmr.2024.100984

**Published:** 2024-07-26

**Authors:** Ali Okan Gazeloglu, Abdurrahman Yilmaz, Egemen Turhan, Filippo Familiari, Gazi Huri

**Affiliations:** a Sincan Training and Research Hospital, Ankara, Turkey; b Department of Orthopaedics and Traumatology, Hacettepe University, Ankara, Turkey; c Department of Orthopaedic and Trauma Surgery, Magna Graecia University of Catanzaro, Catanzaro, Italy

## Abstract

**Purpose:**

To assess the modified 1 anterior portal Bankart repair and compare it to the 2-portal Bankart repair in terms of surgical time, functional scores, and recurrent dislocation.

**Methods:**

Patients who underwent Bankart repair from 2014 to 2021 were identified and separated into 2 groups: a modified 1 anterior portal group and a 2 anterior portal group. The inclusion criteria were being >18 years old, having a recurrent anterior shoulder dislocation with a Bankart lesion, and having a minimum 2-year follow-up. Patients were evaluated for their clinical results using the American Shoulder and Elbow Surgeons score, the Western Ontario Shoulder Instability index, and the Oxford Shoulder Instability Score pre- and postoperatively. The duration of surgery and recurrent instability were recorded. To prevent suture tangling in the modified 1-portal group, 2 techniques were performed: “cannula in cannula” and “cannula in and out.”

**Results:**

A total of 42 patients were included in this study, with 20 in the modified 1-portal group and 22 in the 2-portal group. There were no statistically significant differences between the 2 groups in clinical scores obtained after 2 years of surgery (American Shoulder and Elbow Surgeons score, *P* = .5; Western Ontario Shoulder Instability index, *P* = .22; and Oxford Shoulder Instability Score, *P* = .32). The average surgical duration in the modified 1-portal group (65.7 ± 15.8) was significantly shorter than the average surgery duration in the 2-portal group (81.1 ± 27.2) (*P* = .03). There was no statistically significant difference between the 2 groups for recurrent instability (*P* ≥ .999).

**Conclusions:**

Bankart repair performed through a modified 1 anterior portal technique has a shorter surgical time and similar clinical outcomes as the 2-portal technique.

**Level of Evidence:**

Level III, retrospective cohort study.

The glenohumeral joint is the most commonly dislocated joint in the body, and it is typically anteriorly and inferiorly dislocated. During dislocation, the shoulder’s anterior soft tissue stabilizers, the anterior inferior labrum and the anterior of the inferior glenohumeral band, are injured.^1^, ^2^, ^3^, ^4^, ^5^ Up to 86% of recurrent instability can occur following the initial dislocation, depending on predisposing factors.^6^

In 1923, Bankart syndrome,^7^ which plays a role in recurrent shoulder instability, was described, including the philosophy of Bankart lesion repair and the open surgery technique. Modern arthroscopic techniques with 3 or 4 portals were described subsequently, even though the fundamental Bankart repair principles remained unchanged.^7^, ^8^, ^9^, ^10^ As with all surgical procedures, the minimally invasive approach has been defined in the Bankart repair of the shoulder, and a method utilizing a single anterior portal has emerged.^11^

Few studies have compared the anterior 1-portal arthroscopy and 2-portal arthroscopy techniques for Bankart repair. Compared to the 2-portal technique, the 1-portal technique has benefits and drawbacks. Suture tangling is one of the issues encountered during Bankart repair of a solitary anterior portal. However, tips to prevent suture entanglement are insufficiently described.^12^, ^13^, ^14^, ^15^

The purpose of this study was to assess the modified 1-portal Bankart repair and compare it to the 2-portal Bankart repair in terms of surgical time, functional scores, and recurrent dislocation. We hypothesized that the modified 1-portal Bankart repair technique is comparably effective to the standard 2-portal technique in terms of postoperative functional scores and recurrent instability and that the modified 1-portal technique has a shorter surgical time compared to the standard 2-portal procedure.

## Methods

The study was carried out following the approval of the Non-Invasive Clinical Research Ethics Committee (no: 2022/17-14). Patients who underwent arthroscopic Bankart repair between January 2014 and August 2021 at the authors’ institution were retrospectively identified. Inclusion criteria were being >18 years old, having a clinically traumatic recurrent anterior shoulder dislocation with a Bankart lesion, and having a minimum 2-year postoperative follow-up. Exclusion criteria were patients needing additional procedures like remplissage or open surgery, having a glenoid defect greater than 25%, having undergone surgery due to associated rotator cuff tears or superior labrum anterior-posterior lesions, having atraumatic multidirectional instability, or undergoing revision instability-related shoulder surgery.

All 1 anterior portal arthroscopic Bankart repair (group 1) and standard 2 anterior portal arthroscopic Bankart repair (group 2) surgical procedures were conducted by 2 senior surgeons (G.H. and E.T.) together. Both surgeons had more than 10 years of experience in performing shoulder arthroscopy. We conducted radiologic assessments using computed tomography and magnetic resonance imaging preoperatively.

The study included patients who underwent arthroscopic Bankart repair through a single anterior portal in group 1 and patients who underwent standard 2-portal arthroscopic Bankart repair in group 2. We measured the external rotation and abduction degrees of the operated shoulder using a goniometer during preoperative and postoperative clinical examinations. Patients’ preoperative and postoperative 2-year assessment data included the American Shoulder and Elbow Surgeons (ASES) score, Western Ontario Shoulder Instability (WOSI) index, and Oxford Shoulder Instability Score (OSIS). Ultimately, patients were evaluated for recurring instability within a time frame of 2 years following the surgical procedure.

### Surgical Technique

All patients underwent surgery in the beach-chair position under hypotensive general anesthesia with the assistance of an arthropump. A classic posterior portal was used for imaging. In group 1, a single anterior portal was created 1 cm lateral and 1 cm superior to the coracoid notch under the view of the posterior imaging portal. Following this, a 7.5-mm cannula was inserted with the assistance of a sent spinal guide needle from the outside. In group 2, 2 anterior portals, anterosuperior and anteroinferior, were created, and two 7.5-mm cannulas were inserted into the portals. In all patients, after glenoid preparation and labral release, nondegradable sutures were passed in a lasso-loop fashion, and labral fixation was achieved using a minimum of 3 knotless anchors (Doratek Medical) with diameters of 2.9 mm or 3.5 mm.

**Fig 1:**
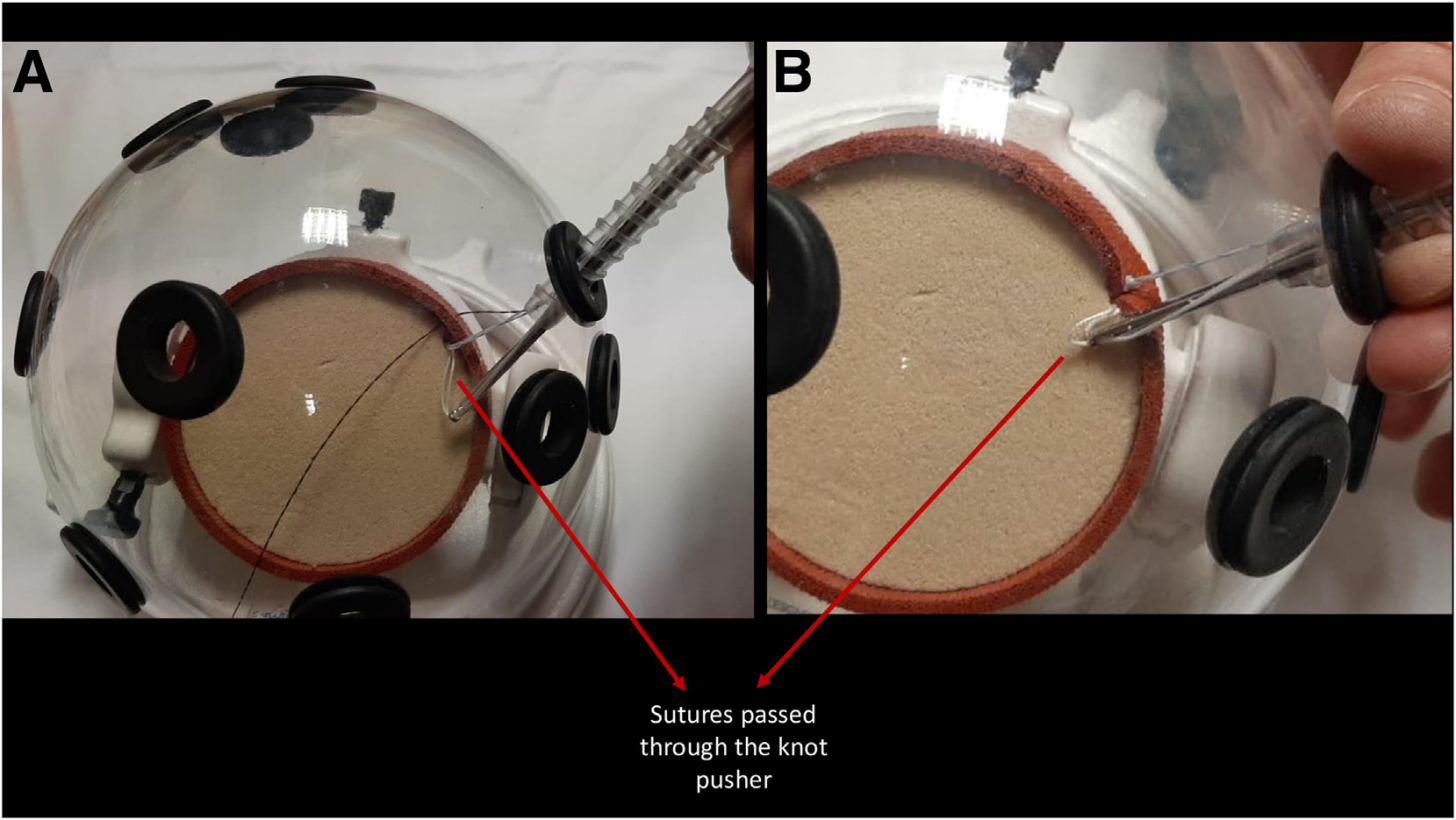
“Cannula in cannula” technique with knot pusher as a second cannula. (A) Before taking a sliding knot in the “cannula in cannula” technique. (B) After taking a sliding knot in the “cannula in cannula” technique.

**Fig 2:**
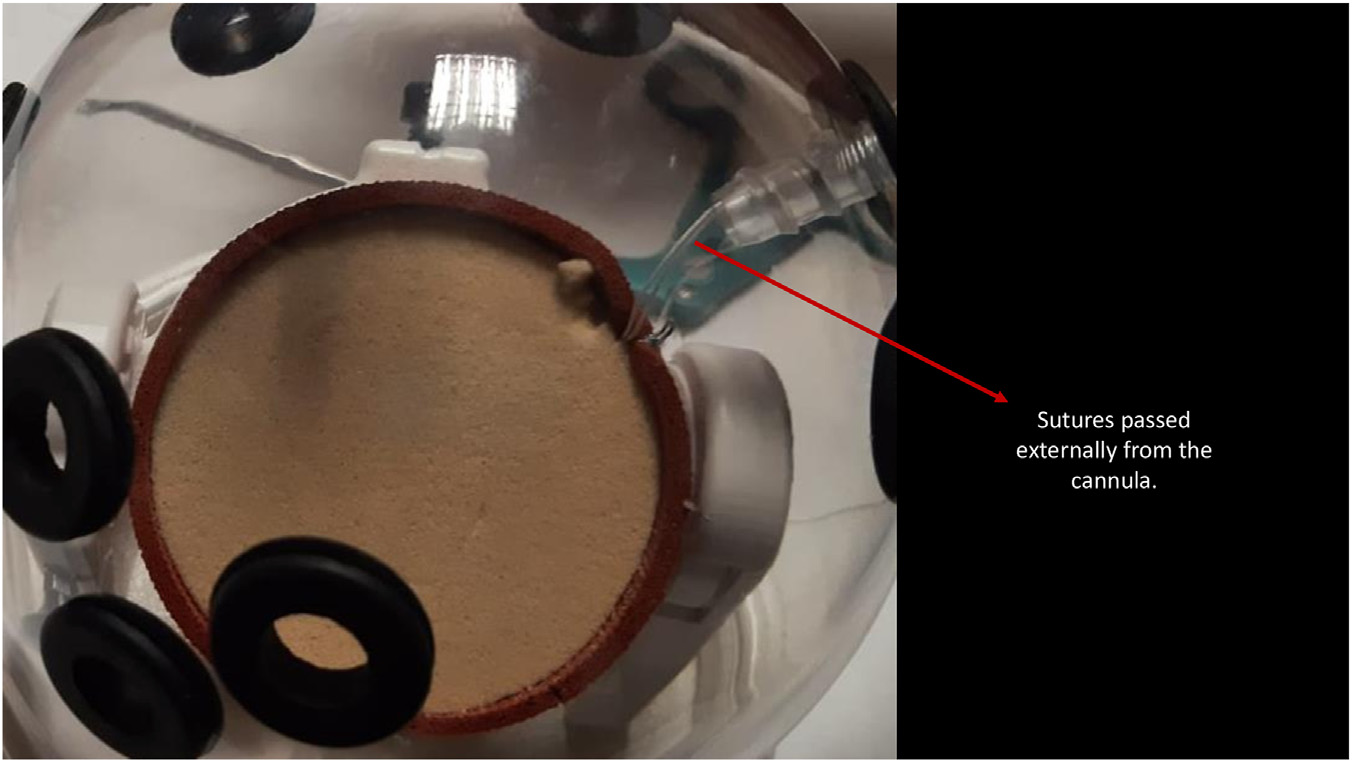
“Cannula in and out” technique. Two of the sutures pass externally from the cannula.

In group 1, 2 techniques were employed to overcome the issue of suture entanglement inherent to this method. The first technique, the “cannula in cannula” technique (Fig 1), involved placing 4 knotless anchors in appropriate positions, then inserting a standard arthroscopy cannula from the anterior portal and pulling the suture through the cannula using a suture manipulator. Subsequently, the sutures were separated into 2 groups of the same color, and the first group was passed through a knot pusher. The knot pusher was then sent through the cannula from the implantation site of the anchor, and the second group of sutures was separated from the implantation area of the anchor. This prevented the knots from the second group from interfering with the knots from the first group. The knot pusher acted as a second cannula in this case. The second technique, the “cannula in and out” technique (Fig 2, Video 1), involved passing only 1 of the 4 knotless anchor sutures through the cannula, while the remaining 3 sutures were passed externally and from the anterior portal using a suture manipulator. Since the sutures were taken individually into the cannula for each surgical step, the problem of suture entanglement was avoided.

Codman’s pendulum exercises were initiated on the first day after surgery. Patients used a shoulder-arm sling with an abduction pillow for 6 weeks after surgery. The rehabilitation program with the Department of Physical Therapy and Rehabilitation began at the end of the first week, with forced external rotation being restricted for 6 weeks. All patients had bimonthly follow-up appointments in the first month, followed by assessments at 3, 6, and 12 months in the first year, and subsequently on an annual basis. External rotation and abduction angles of the operated shoulder were recorded during these assessments. Any perioperative or postoperative complications were also documented.

### Statistical Analysis

G∗Power software (Windows version 3.1.9.7; Heinrich-Heine-Universität Düsseldorf) was used to execute a power analysis to determine the sample sizes for both groups. The effect size was calculated to be 1.42 in a study conducted by Uzun et al.^13^ by using the variable of operating time, which was a similar study in the literature. To exceed the 80% value for the study’s power, at a significance level of 5% and an effect size of 1.42, at least 7 patients were required in each study group.

To analyze statistical data, IBM SPSS (Windows version 23.0; IBM Corp.) was utilized. Skewness and kurtosis values, histogram analysis, and Kolmogorov-Smirnov test outcomes were used to determine whether the data had a normal distribution. The descriptive data were expressed as mean plus standard deviation, with minimum and maximum values also provided.

Independent numerical data were analyzed using the *t* test of independent variables and the Mann-Whitney *U* test. Using the Wilcoxon signed rank test, quantitative variables in dependent groups were analyzed statistically. For the analysis of nominal data for independent groups, the χ^2^ test was utilized. The Fisher exact test was used when the χ^2^ test indicated that the expected values were less than 5. *P* < .05 was considered statistically significant.

## Results

**Fig 3:**
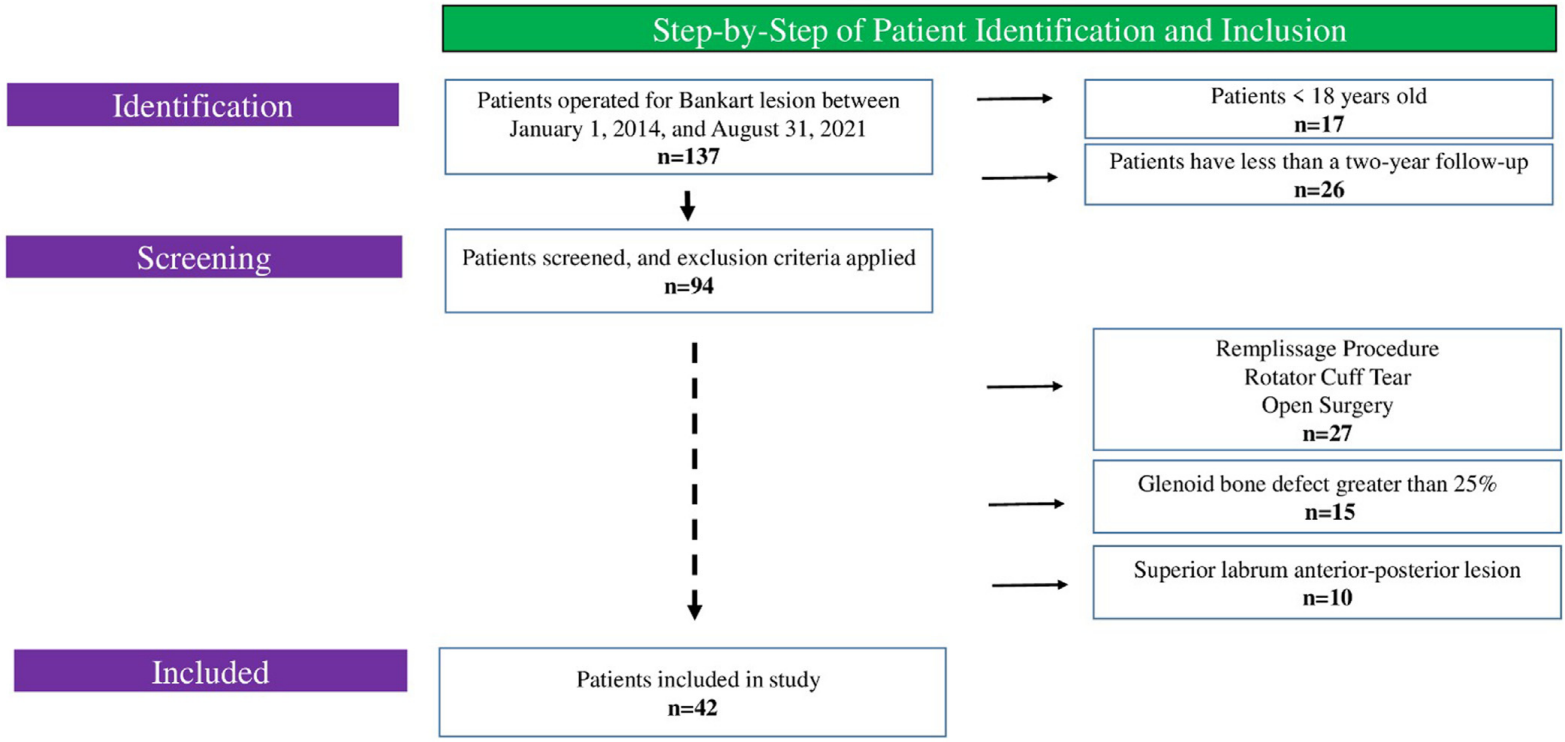
The flowchart for patient selection.

A total of 42 patients met the inclusion criteria (Fig 3). The average age was 25.9 years (range, 19-31 years). There were 20 patients in the 1 anterior portal group (group 1) and 22 in the standard 2-portal group (group 2). Table 1 shows the demographic information of the patients.

**Table 1:** Patients’ Descriptive Statistics

Characteristic	Total (N = 42)	Group 1 (n = 20)	Group 2 (n = 22)	*P* Value
Sex, female/male, n	3/39	1/19	2/20	**≥.999**
Age, mean ± SD, y	25.9 ± 5	25.9 ± 5.5	26 ± 4.6	**.97**
Affected side, right/left, n	13/42	8/12	5/17	**.22**
Number of preoperative dislocations, mean ± SD	1.8 ± 0.9	1.9 ± 0.9	1.7 ± 0.9	**.54**
Length of hospital stay, mean ± SD, d	1.04 ± 0.2	1.1 ± 0.3	1 ± 0	**.13**
Follow-up time, mean ± SD, mo	**51.2 ± 22.1**	**44.2 ± 20.5**	**57.5 ± 22.1**	.05

NOTE. Group 1 indicates patients treated with the modified 1-portal technique, and group 2 indicates patients treated with the standard 2-portal technique. *P* < .05 is statistically significant. Bold values are statistically significant.

Both groups’ preoperative and postoperative clinical evaluations of the second year were evaluated. Statistically, there was a significant improvement in ASES score, WOSI index, and OSIS postoperatively in both modified 1-portal and 2-portal surgical treatments (*P* < .001). Comparing the clinical scores of the groups in the second year postoperatively revealed no statistically significant differences between the ASES score (*P* = .5), WOSI index (*P* = .22), and OSIS (*P* = .32). The clinical assessments for both groups are detailed in Table 2.

**Table 2:** Patients’ Clinical Assessments

Characteristic	Modified 1-Portal Group			Two-Portal Group			Preoperative *P* Value∗	Postoperative *P* Value∗
	Preoperative, Mean ± SD	Postoperative, Mean ± SD	*P* Value†	Preoperative, Mean ± SD	Postoperative, Mean ± SD	*P* Value†		
Clinical scores								
ASES score	57.8 ± 16.6	96.2 ± 7.1	<.0001	59.5 ± 9.7	98.1 ± 2.3	<.0001	**.87**	**.5**
WOSI index	53.4 ± 14.3	5.7 ± 6.9	<.0001	53.9 ± 12.8	3.2 ± 2.7	<.0001	**.61**	**.22**
OSIS	25 ± 5.5	46.4 ± 3.9	<.0001	23.9 ± 7.3	47.2 ± 1.2	<.0001	**.45**	**.32**
Range of motion								
Abduction (angle)	NA	168° ± 13°	NA	NA	171° ± 12°	NA	NA	**.33**
External rotation (angle)	NA	85° ± 4°	NA	NA	85° ± 5°	NA	NA	**.86**

NOTE. *P* < .05 is statistically significant. Bold values are statistically significant.

ASES, American Shoulder and Elbow Surgeons; NA, not applicable; OSIS, Oxford Shoulder Instability Score; WOSI, Western Ontario Shoulder Instability.

∗ Compared between 2 groups’ values.

† Compared within each group for their preoperative and postoperative scores.

The range of motion of the patients’ shoulder joint was assessed. In the modified 1-portal technique, the average postoperative second-year abduction angle was 168° ± 13°, whereas in the 2-portal technique, it was 172° ± 12°. In the modified 1-portal technique, the average postoperative second-year external rotation angle measurement was 85.5° ± 5°, whereas it was 85.2° ± 6° in the 2-portal technique. In the second year, there was no statistically significant difference in abduction and external rotation measurements between the 2 groups (*P* = .33 and *P* = .86, respectively).

Both groups’ durations of surgery (Table 3) were compared. Statistically, the average surgical duration for patients in group 1 was significantly shorter than the average surgeon duration for patients in group 2 (*P* = .03).

**Table 3:** Comparison of the Groups in Terms of Duration of Surgery and Recurrent Instability

Characteristic	Modified 1-Portal Group	Two-Portal Group	*P* Value
Duration of surgery, mean ± SD, min	65.7 ± 15.8	81.1 ± 27.2	**.03**
Recurrent instability, % (n)	5 (1/20)	4.5 (1/22)	**≥.999**

NOTE. *P* < .05 is statistically significant. Bold values are statistically significant.

Two patients experienced recurrent instability after Bankart repair, and there was no statistically significant difference between the 2 groups in terms of postoperative recurrent instability (*P* ≥ .999).

## Discussion

The findings obtained in our study have demonstrated that Bankart repair performed through a single anterior working portal is effective, reliable, and reproducible. Additionally, Bankart repair performed through the standard 2 working portals yields similar clinical outcomes in terms of functional results.

One-portal arthroscopic Bankart repair has become increasingly popular in the past 5 years due to its less invasive nature.^11^, ^12^, ^13^^,^^16^, ^17^, ^18^, ^19^ In our study, there was a statistically significant improvement in clinical scores within both the 1-portal and the 2-portal groups. There was no significant difference between the 2 groups. Similarly, both groups yielded similar results in terms of external rotation and abduction degrees during the 2-year follow-up examinations. There was no difference between the groups in terms of recurrent dislocations. In addition to having similar benefits, the 1-portal group exhibited a significant decrease in surgical duration. It also has advantages such as less surgical scarring, lower risk of neurovascular damage, and lower cost due to the use of fewer materials.^12^

Few studies have compared the outcomes of 1-portal and 2-portal arthroscopic Bankart repairs. Çiçek et al.^12^ showed that the 1-portal group had less postoperative pain, had a shorter learning curve for surgery, and was more cost-effective compared to the 2-portal group. The average surgical duration in the 2-portal group was 53 minutes, while it was 35 minutes in the 1-portal group (*P* < .001). Although clinical outcomes were similar between the groups, the 1-portal technique was considered to better protect the patient from instability due to its less invasive nature. Ghai et al.^20^ also obtained similar Rowe scores, OSIS, and Tegner activity levels in both 1- and 2-portal groups. However, as shown in our study, the 1-portal technique was proven to be significantly superior in terms of surgical duration. Armangil et al.^16^ emphasized that both 1-portal and 2-portal groups had similar clinical outcomes, but the 1-portal group had shorter surgical durations and was less invasive. Uzun et al.^13^ retrospectively evaluated 1-portal and 2-portal patients and, similarly, found no significant difference in functional scores but considered the 1-portal group advantageous in terms of surgical duration. In the study by Sebastiá-Forcada et al.,^17^ which evaluated prospectively 1-portal and retrospectively 2-portal patients, successful results were achieved in both groups except for SLAP type 3 patients, and it was stated that the 1-portal group had shorter surgical durations and was a cost-effective treatment.

There is a nonnegligible risk of complications during arthroscopic Bankart repair, including portal placement, anchor insertion, and labral repair processes. The 2-portal technique can increase the risk of neurovascular damage in individuals with a small shoulder. In these patients, the lack of sufficient space for double cannula placement can make surgical repair difficult and increase the risks of cannula breakage and nerve damage.^13^^,^^14^^,^^21^^,^^22^ In our study, no serious complications were detected during surgery or follow-up.

### Limitations

The study has some limitations. First, the retrospective nature of this study is prone to bias and confounding. Second, the relatively small number of patients creates the potential for a type II error. Third, the hospital stay duration was not compared between the groups. This may have been an important variable to measure regarding immediate postoperative results. Finally, we did not perform a cost analysis between the groups, so we could not report on the financial aspect of the techniques.

## Conclusions

Bankart repair performed through a modified 1 anterior portal technique has a shorter surgical time and similar clinical outcomes as the 2-portal technique.

## Disclosures

All authors (A.O.G., A.Y., E.T., F.F., G.H.) declare that they have no known competing financial interests or personal relationships that could have appeared to influence the work reported in this paper.

## Supplementary Data

Video 1 The demonstration of the “cannula in and out” technique.
